# Retorting conditions affect palatability and physical characteristics of canned cat food

**DOI:** 10.1017/jns.2017.17

**Published:** 2017-05-22

**Authors:** Esther A. Hagen-Plantinga, Denmark F. Orlanes, Guido Bosch, Wouter H. Hendriks, Antonius F. B. van der Poel

**Affiliations:** 1 Department of Nutrition, Faculty of Veterinary Medicine, Utrecht University, Utrecht, The Netherlands; 2 Animal Nutrition Group, Wageningen University, Wageningen, The Netherlands

**Keywords:** Thermal processing, Palatability, Texture, Viscosity, Canned food, Cats, *F*_0_, lethality value

## Abstract

The effects of different temperature and time conditions during retorting of canned cat food on physicochemical characteristics and palatability were examined. For this purpose, lacquer cans containing an unprocessed loaf-type commercial cat food were heated in a pressurised retorting system at three specified temperature–time profiles (113°C/232 min, 120°C/103 min and 127°C/60 min) to equal a similar lethality (*F*_0_ value = 30). Physicochemical properties (viscosity, texture, particle size, pH) were determined, and a 10 d three-bowl palatability test was performed with ten European shorthair cats. Retorting at 113°C/232 min resulted in differences in all the physical parameters examined (<viscosity, firmness, adhesiveness, and > particle size). Significant pH differences were observed (6·53, 6·63 and 6·66 for T113/232, 120 and 127°C, respectively). Preference ratios were 0·38, 0·31 and 0·31 for T113/232, 120 and 127°C, respectively (*P* = 0·067). It can be concluded that different retorting temperature–time profiles with equal *F*_0_ value significantly affect physical characteristics and tended to affect palatability of moist cat food.

Moist commercial feline foods are manufactured using retort processing to sterilise and preserve the product and to obtain a desired consistency. During retorting, food contents are sealed in airtight cans, containers or flexible pouches and are subsequently heat treated^(^^1^^)^. The effectiveness of a heating process in achieving product sterility can be evaluated by determination of the lethality value (*F*_0_) of the process. The *F*_0_ represents the time equivalent of a heating process to destroy micro-organisms at the reference temperature of 121·1°C and serves as a standard to compare sterilisation values for different processes^(^^2^^)^. Any process which achieves an *F*_0_ of 3 min is considered to render the product free from pathogenic organisms, which could create a public health danger^(^^3^^)^. In pet food production, however, higher values (*F*_0_ values > 10) are employed in order to destroy bacterial spores as well^(^^2^^)^. Besides sterility of a product, additional factors related to the product quality such as physicochemical factors and palatability may be affected by the retorting conditions.

Little is published in the scientific domain on effects of retorting conditions on physicochemical factors of canned pet food. Niamnuy & Devahastin^(^^4^^)^ stated that excessive heating temperature and time may lead to overcooking and lead to undesirable textural characteristics of a product, as this may affect the binding properties of the product. As texture is in close relationship with palatability (as chewiness, hardness and elasticity affect mouth feel of the product), excessive heating may also negatively affect palatability. With regard to palatability, under high temperature and pressure of retorting, desirable flavour compounds, like sulphur-containing thiazole and thiopene compounds, can develop as a result of occurrence of the Maillard reaction between free reactive amino groups of specific amino acids and reducing sugars^(^^5^^)^. On the other hand, Heinicke^(^^6^^)^ reported that an increasing retorting time may negatively influence palatability for cats, which may be attributed to formation of lipid peroxides, and/or more aroma-intensive heterocyclic Maillard reaction products, such as pyrrole, pyridine and pyrazine, giving undesirable flavours during retorting^(^^4^^)^.

The present study aimed to examine the effects of three different temperature/time conditions during retorting of canned cat foods on nutritional and physicochemical characteristics and palatability in cats. These conditions were chosen to reflect specific practical goals in the retorting process: to maintain nutritional value of the product (e.g. reactive lysine content) (113°C/232 min; T113/232); to minimise energy consumption of the retort process (120°C/103 min; T120/103); and achieve a short processing time and high plant capacity (127°C/60 min; T127/60). Even though the effects of retorting on physicochemical factors and palatability have been reported in the literature^(^^2^^,^^6^^)^, those studies mainly measure the effect of increasing *F*_0_ values by increasing the retorting time. To the authors’ knowledge, no studies have been conducted that investigated the effects of different retorting temperature–time profiles while employing an equal *F*_0_ value on different quality parameters of cat food.

## Materials and methods

### Preparation of diets

Lacquer cans (400 g, diameter 72 mm, height 10·4 cm) containing a raw mixture of a loaf-type commercial cat food were heated in a pressurised retorting system at three specified temperatures (113, 120 and 127°C) and at corresponding processing times (232, 103 and 60 min, respectively) to reach a similar *F*_0_ value of 30.

A vertical autoclave (Veerman B.V. Autoclave) with a capacity to hold 180 cans in one layer was used. For each retorting batch (180 cans per combination of time/temperature), a data logger (Pico Vacq PT, TMI Orion) was placed in the geometric centre of a can that was placed in the middle of the autoclave. Temperature and pressure were recorded at 12 s intervals during retorting.

The execution of retorting comprised three phases: phase 1 of heating up, when the temperature of the autoclave was brought to the desired temperature; phase 2 of holding, when the retorting temperature was achieved and maintained to obtain the target *F*_0_ value; and phase 3 of cooling, when water was introduced in the autoclave to cool down the product. For T113/232, these phases had a duration of 23·8 (phase 1), 232·0 (phase 2) and 5·5 (phase 3) min, for T120/103 these were 27·9 (phase 1), 103·0 (phase 2) and 5·5 (phase 3) min and for T127/60 the phases lasted 35·7 (phase 1), 60·0 (phase 2) and 6·5 (phase 3) min. Other processing factors in the retorting process, like pressure and humidity, were kept as constant as possible, to assure that differences between the different diets could indeed be attributed to variation in time and temperature. The stored temperature and pressure data from the data logger were used to calculate the concomitant *F*_0_ values of each batch of cans.

### Chemical analyses

The retorted foods were analysed for DM by drying the samples at 103°C to constant weight and crude ash by combustion at 550°C for 3 h. The crude fat content was analysed according to the Berntop method^(^^7^^)^ and total N using the Kjeldahl method^(^^8^^)^. Crude protein was calculated as total N × 6·25. The pH of the diet was measured in duplicate per can, using three cans per diet by inserting the probe into the middle of the can.

### Viscosity measurements

For each diet, three cans were randomly selected and processed for viscosity measurement. The content of each can was manually mixed for 1·5 min. A representative sample (7 g) was centrifuged at 3000 ***g*** for 10 min at 25°C to separate the solid and fluid portions of the food. The apparent viscosity of the fluid was measured in duplicate for each can using 1 ml fluid sample in a Brookfield viscometer DV-II (Brookfield Engineering Laboratories) with spindle CP-40 at 25°C and shear rate of 150/s.

### Texture analysis

A modified back extrusion test^(^^9^^)^ was employed to determine food texture using three samples per can and three cans per diet. A texture analyser (TA.HD plus; Mason Technology) equipped with a 50 mm diameter probe was used as a plunger with the 72 mm-diameter tin can as the cell. The space between the plunger and tin can served as the annulus where the food was allowed to flow through. The plunger was gently pushed into a can at the rate of 1 mm/s with a maximum load of 5 kg and was set to penetrate the food 2 cm deep. Data on the force applied was deduced to determine the firmness, adhesiveness and chewing work of the product. Firmness (maximum force to maintain extrusion; kg) provides an indication of the force required to press the food during mastication. Chewing work (kg × s) is the area of the positive force curve that indicates total effort on chewing the food^(^^10^^)^. Adhesiveness (kg) is the maximum negative force when the plunger is withdrawn which indicates the stickiness of food^(^^10^^)^.

### Particle size determination

The particle size distribution was determined by wet sieving analysis using a Retsch apparatus AS-200 (Retsch BV). Two 50 g aliquots from two cans of each of the three diets were placed on the top of a sieve apparatus which was equipped with six sieves (2·5, 1·25, 0·63, 0·315, 0·160 and 0·071 mm openings) and a solid pan at the bottom. Water was introduced to fill the sieves while shaking for 5 min after which the water was drained and the sieves shaken for another 10 min. Filling and draining were repeated three times after which the sieves were shaken for a final 45 min. Samples were collected from each screen and their DM weights determined and used to calculate the geometric mean diameter and its geometric standard deviation as described previously^(^^11^^)^.

### Palatability testing

A total of ten domestic short-hair cats (one neutered male and nine neutered females, aged between 5·0 and 7·3 years) with a body weight ranging from 2·6 to 5·7 kg were used in a 10 d, three-bowl palatability test. The cats were housed individually in cages (75 × 75 × 75 cm^3^) for 16 h/d at the De Morgenstond animal testing facility (Dussen, the Netherlands). All cats had access to a communal play area for 8 h per d during which time cats did not have access to food.

During the palatability test, cats were provided with three bowls containing a pre-weighed amount (400 g) of each diet in separate bowls. Each cat's first choice was recorded. First choice was determined during the first 2 min after cats were placed in the cage by the handler to start the consumption of food. A mark of +3 was given to the first diet consumed, and −3 to the diets not selected within those 2 min. A zero value was given to the diets when no clear preference was noticed within the first 2 min after the cat was placed in the cage. The placement of bowls in the cage was changed daily (e.g. A–B–C, C–A–B, B–C–A) throughout the 10 d of testing. At the end of the 16 h in-cage period, bowls were collected and leftover foods were weighed. Food intake of cats was expressed as a modification on the intake ratio (in %) as described by Crane *et al*.^(^^1^^)^: A/(A + B + C) × 100, where A, B and C are individual daily food consumption of each of the three different foods.

Approval of the Ethical and Welfare Committee was sought, but not required under the Dutch law, as the design was non-invasive in nature.

### Data analyses

Differences in diet preference were tested using a related-samples Friedman's two-way ANOVA (SPSS 24.0.0.1; IBM). Differences in physicochemical parameters (viscosity and texture) were tested using a one-way ANOVA with Bonferroni-adapted *P* values. A significance level of 0·05 was used in the analysis. Pearson correlations between cat food preference and physical parameters were obtained by correlation analyses of SPSS 19. The first choice scores were summed for each cat and means were compared through ANOVA testing for each diet.

## Results

The deduced *F*_0_ values for T113 and T120 were 30 but for T127 this was 39. Nonetheless, an *F*_0_ value of 39 is within the *F*_0_ value range of commercial retorting for loaf-type cat food.

### Chemical analyses

The DM content of the cat food was 230 g/kg and the crude protein, crude fat and ash contents were 468, 274 and 141 g/kg DM, respectively.

### Physicochemical analyses

Cat foods retorted at 113°C/232 min differed from those retorted at 120°C/103 min and 127°C/60 min for all the physical parameters examined (<viscosity, firmness, adhesiveness, and >particle size; *P* < 0·05) (Table 1). Furthermore, pH was lower when retorting at 113°C than for the other two retorting treatments (*P* < 0·0·05).

**Table 1.** Physical and chemical parameters of canned cat foods processed using three retorting conditions (Mean values with their standard errors)

	113°C/232 min		120°C/103 min		127°C/60 min	
Parameter	Mean	sem	Mean	sem	Mean	sem
GMD (μm)	620^a^		470^b^		441^b^	
GSD	65·8		30·4		15·6	
Viscosity (mPa)	3·3^a^	0·04	7·9^b^	0·07	7·3^b^	0·08
Texture						
Firmness (kg)	1·99^a^	0·15	3·51^b^	0·28	4·01^b^	0·12
Adhesiveness (kg)	0·22^a^	0·06	0·42^b^	0·03	0·42^b^	0·02
Chewing work (kg × s)	24·0^a^	4·1	53·9^b^	9·9	59·3^b^	2·2
pH	6·53^a^	0·00	6·63^b^	0·00	6·66^b^	0·01

GMD, geometric mean diameter based on wet sieving; GSD, geometric standard deviation.

^a,b^ Values with unlike superscript letters were significantly different (*P* < 0·05).

### Palatability/food preference

**Fig. 1.**
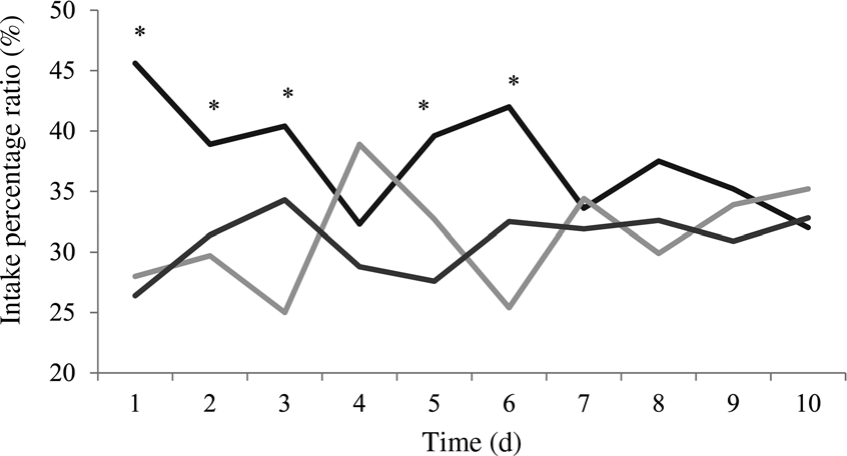
Intake preference of cats (*n* 10) provided with canned cat foods processed with the three retorting conditions: 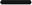, 113°C/232 min; 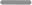, 120°C/103 min; 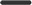, 127°C/60 min. * Mean values for the different conditions were significantly different (*P* < 0·05).

All cats accepted the diets throughout the testing days. Food preferences were compared on a daily basis (Fig. 1). Overall preference ratios were 0·38, 0·31 and 0·31 for T113/232, T120/103 and T127/60, respectively. There was a tendency towards a significant difference in preference (*P* = 0·067) between T113/232 *v*. T120/103 and T127/60. There was no significant difference between T120/103 and T127/60 on the overall percentage intake. Cats significantly preferred T113/232 for the first 6 d except day 4 at which T120/103 and T113/232 were preferred equally. Indifference was observed from day 7 to day 10.

The first choice scores of cats showed no significant differences between T113/232 and T127/60 (mean score = −8·7 and −2·1, respectively; *P* = 0·190). Also, no differences were observed between T113/232 and T120/103 (mean score = −15; *P* = 0·210). T127/60, however, showed a significantly higher first choice score compared with T120/103 (*P* = 0·014).

The results of the first choice test were not correlated with which food was ultimately preferred by the cats. Only 16·2 % of cats that first chose T127/60 ultimately showed preference for T127/60, and 60 % of cats that first chose T127/60 ultimately preferred to eat T113/232. Among the cats that first chose T113/232, 50 % preferred to eat T113/232 and only 19·2 % preferred T120/103. Additionally, 37·5, 31·2 and 31·2 % of the cats that first chose T120/103 ultimately preferred to eat T113/232, T120/103 and T127/60, respectively. Bowl or side bias was not observed in the test.

### Correlation analysis

In the correlation analysis, pH (*r* −0·824; *P* = 0·006), firmness (*r* −0·721; *P* = 0·028) and viscosity (*r* −0·751; *P* = 0·000) were negatively correlated with food intake (measured as intake percentage) of the cats. The physical parameters geometric mean diameter (*r* 0·774; *P* = 0·71), adhesiveness (*r* −0·645; *P* = 0·061), elasticity (*r* −0·455; *P* = 0·219) and chewing work (*r* −0·629; *P* = 0·07) did not have a statistically significant correlation with the intake percentage of cats. Moreover, the first choice score of cats did not have significant correlation with the percentage of intake of cats (*r* 138; *P* = 0·469).

## Discussion

Retorting is an intense heating process to sterilise food. It may affect nutrient bioavailability and palatability of loaf-type cat food^(^^2^^,^^6^^)^. In this study, its effects on palatability were assessed through preference testing and physical parameter analysis that may indicate textual perception.

Physicochemical parameters were different when retorting at lower temperature (113°C) and longer time (232 min) compared with the other two retorting treatments. The decrease in viscosity of the food can be due to the decrease in water-binding capacity of processed meat. Meat processed at higher retorting temperature has been shown to retain its water-binding capacity to a greater degree than meat processed at a lower temperature when both of the products were processed at the same *F*_0_ value^(^^12^^)^. The increase in unbound water was shown to dilute other fluids; thus a reduction in viscosity was observed. A similar explanation can be associated with the decrease in firmness, chewing work and adhesiveness of the food. The increase in free water may interfere in the protein matrix that weakens the binding quality of the particles^(^^12^^)^, hence decreasing the firmness and stickiness of the diet. Additionally, the particle size and particle distribution were also significantly different. T113/232 showed a larger average particle size and larger particle size distribution.

Apart from physicochemical analysis, the diets were also tested on animals for their palatability. The results showed an overall tendency to preference for T113/232, with a significant preference from days 1 to 6 with an exception on day 4. Indifference on intake percentage was observed from days 7 to 10.

The first choice test showed that cats have the tendency to select T127/60 as a first choice, and was not correlated with which food was ultimately preferred by the cats. The results suggest that in this study the first choice does not represent the overall sensory perception of the food using the three-bowl test. Pickering^(^^13^^)^ mentioned that it is flavour rather than the colour and ortho-nasal aroma that is dominant in influencing the food preference of cats.

Pearson correlations were performed between the preference results and the physical parameters to interpret the physical and chemical parameters of the food in terms of sensory perception. Negative correlations with diet preference were only significant for pH, firmness and viscosity of the cat food. The higher palatability of T113/232 might thus, in part, be related to the lower pH of this diet, as this may result in more efficient stimulation of the abundant acid unit taste receptors of cats^(^^14^^)^. In addition, the longer retorting time probably promoted the formation of more and/or different aromatic compounds, which can positively influence food palatability in cats^(^^15^^)^. Moreover, Bradshaw *et al*.^(^^16^^)^ mentioned that ‘bitter amino acids’ like l-arginine tend to be avoided by cats. Consequently, loss of l-arginine during Maillard reactions may increase the palatability of the diet. The negative correlation between viscosity and preference may be linked to flavour release. The intensity of flavour release was observed to be negatively affected by the increasing viscosity of the product^(^^17^^)^. To determine whether it is the flavour chemicals or textual sensation that contributes to the preference values, further studies on the profile and kinetics of volatile compounds present in the diets are needed.

Adhesiveness, chewing work and particle size were significantly different for T113/232 from the other diets, but did not correlate with the results from the preference study. This is in line with the observations in piglets of Sola-Oriol *et al.*^(^^10^^)^, showing absence of correlation between adhesiveness and preference. The effect of saliva production, which acts as a lubricant, may dampen the stickiness of the diet, thus reducing the effect of the adhesive characteristic of the diet. For chewing work, a negative correlation with preference values was reported by same author. In our study, however, no significant correlation was observed between chewing work and preference values. A possible explanation for this discrepancy might be that cats do not need to chew much when eating canned food. Indeed, Watanabe *et al*.^(^^18^^)^ found that cats open their mouths less actively and at a higher pace when eating canned food, meaning that little or no actual chewing is performed^(^^19^^)^. The correlation between particle size and sensory perception was not statistically significant (*P* = 0·076), which is in accordance with earlier works on piglets^(^^12^^)^.

### Conclusion

Different retorting conditions affected the physicochemical characteristics of cat food (pH, firmness and viscosity), which in turn tended to change the initial preference of the animal. The diet processed at the lower temperature and for a longer time was preferred compared with the other diets.
